# Dr. Marshall, on the Cow-Pox

**Published:** 1800-12

**Authors:** J. H. Marshall


### *Dr. Marshall, on the Cow-Pox.*

Copy of a letter from Dr. MARSHALL to Mr. RING, dated Mahon, Iſland of Minorca, September 1800.

MY DEAR SIR,

IN my letter from Gibraltar, I informed you of our intention to proceed to this place. We arrived here upon the 7th inſt, and have been buſily employed amongſt the Navy and Army, who are both equally anxious to avail themſelves of the excellent diſcovery of our friend JENNER. The inhabitants here, from the great dread they entertained of the Small-pox, were cautious reſpecting the Cow-pox; but were ſo convinced of its innocency, from our arguments, as to permit us to inoculate ſome of the infants in the Foundling Hoſpital. From its mild effects upon them, we have now been ſolicited to extend the practice to their own children; and if it becomes general, as it probably will, we ſhall be detained here a few weeks longer.—From hence we intend proceeding to Palermo, Malta, or Naples, as a conveyance may offer; and I have to beg the favour of you to ſend me ſome Cow-pock virus to Naples and Palermo.

I am ſorry that the great haſte in which I am obliged to cloſe my letter, prevents the poſſibility of my adding more, than that the Cow-pox, in every inſtance, preſerves its characteriſtic mildneſs. I remain, &c.

J. H. MARSHALL.

---

Copy of a letter from Dr. MARSHALL to Mr. RING, dated H. M. S. Foudroyant, Gibraltar Bay, Oct. 16, 1800.

DEAR SIR,

YOU, no doubt, will be ſurpriſed at receiving a letter from me, dated Gibraltar, ſo ſpeedily after the one I did myſelf the pleaſure of writing to you from Mahon; but as, upon our arrival there, we were too late to join Lord Keith, he having failed the week previous, I determined to embrace an opportunity that offered of joining him, which I did yeſterday.

He has received me with great politeneſs, and not only permitted but recommended the inoculating of the Fleet for the Cow-pox; and I this day commence. I am now, and ſhall continue, on board his ſhip, till an opportunity occurs of going up to Palermo or Naples, which, I underſtand, will be in a very ſhort time.

I am

I am happy to inform you, that the Cow-pox is in full practice here amongſt the inhabitants, and has preſerved all its uſual mild characteriſtics in every inſtance. It was with great pleaſure as well as ſurpriſe, that my former friends received me again; and the gratitude they expreſs to our friend Jenner, for the benefit they experience from his great diſcovery, muſt be gratifying to all his friends. Dr. Walker I left buſy at Minorca; from whence he will proceed up to Malta, and we ſhall join again at Palermo.

I muſt beg a frequent ſupply from you of the virus, as I wiſh to try as many experiments as I can upon the activity of it, at different ages. I am ſorry to inform you, that what I procured from you, and put up ſo carefully, is now grown inactive; however, that is now four months old.

The troops that are here in the expedition, are falling down very faſt in the ſcurvy; and are in general ſickly. At Cadiz they have had a dreadful diſorder; which, ſo far as I can make out from the report of the phyſician who was ſent there from Madrid, and which I have ſeen, has every character of the yellow fever, attended with bilious vomiting, great proſtration of ſtrength, feeble pulſe, delirium, &c. and is highly infectious. Every precaution is uſed here, to prevent any communication with Spain; though I underſtand, that ſince the commencement of the rains, its violence has greatly abated.

I remain, &c.

J. H. MARSHALL.

